# Farmers’ willingness to adopt geographical indication practice in Indonesia: A psycho behavioral analysis

**DOI:** 10.1016/j.heliyon.2022.e10178

**Published:** 2022-08-11

**Authors:** Pandu Laksono, Irham, Jangkung Handoyo Mulyo, Any Suryantini

**Affiliations:** a Doctoral Student in Agribusiness Management, Faculty of Agriculture, Universitas Gadjah Mada, Yogyakarta, Indonesia; b National Research and Innovation Agency Republic of Indonesia, Indonesia; c Department of Agricultural Socioeconomics, Faculty of Agriculture, Universitas Gadjah Mada, Yogyakarta, Indonesia

**Keywords:** Geographical indication coffee, Willingness to adopt, Structural equation model, Theory of planned behavior, Technology acceptance model

## Abstract

The paper examined the factors influencing farmer’s willingness to adopt GI (geographical indication) practices in the Indonesian coffee sector from a psycho behavioral perspective. Specifically, the paper examined the psychological factors influencing the willingness of farmers to adopt GI. The study combined (1) the Planned Behavior (TPB) theory and (2) Technology Acceptance Model (TAM) as the theoretical framework. The following psycho behavioral factors were constructed and tested: subjective norm (SN), perceived behavioral control (PBC), attitudes toward behavior (ATB), perceived usefulness (PU) and perceived economic benefit (PEB). The study also investigated the effects of sociodemographic factors on these psycho behavioral constructs. The survey was conducted in two geographical indication coffee territories in Indonesia that involved 178 farmers who are perceived as willing to adopt GI practices and procedures. The relationship between constructs was investigated in which structural equation modeling (SEM) was used. The obtain data were analyzed using WarpPLS 7.0. The study finds that attitude toward behavior, perceived behavioral control, and perceived economic benefit, as important factors influencing the willingness to adopt GI practices. The subjective norm did not affect willingness to adopt GI practices. Farmers’ knowledge mainly affected perceived behavioral control and willingness to adopt GI practices and procedures.

## 1 Introduction

Many researches have been conducted on the influence of coffee certification on farmers’ socioeconomic conditions and also factors that affect their willingness to accept the certification schemes (Chiputwa et al., 2015; Jena et al., 2017; Neilson et al., 2018; Vellema et al., 2015). Chiputwa et al. (2015) investigated the influence of production and certification requirements on smallholders' livelihoods in Uganda. By using the choice modeling method, this study evaluates the effects of three different types of certifications of coffee farmers: fair trade, UTZ, and organic. The findings revealed that the three certification methods in place had an impact on farmers' living standards, which were higher than those of farmers who did not engage in the program. Jena et al. (2017) reported that fair trade and organic coffee certifications had differing effects on farmers participating in the certification program: fair trade farmers gain from increased crop yields, whereas organic farmers benefit from increased coffee selling prices. Velema et al. (2015) also looked at the impact of specialty coffee certification on Colombian family livelihoods. According to the study’s findings, certification promotes the coffee farmers to concentrate in high quality production, increasing farm income although not total household income, especially in the long run. Farmers will have to halt other activities due to the extra time and effort they devote to fulfill certified production standards. The fact that certified coffee production does not affect overall family income implies that the return on additional work for certification process is not bigger than in other activity. Certification is commonly followed by the establishment of provisions and standards of practices (Levy et al., 2016), and provides a set of principles to which certified suppliers are required to adhere, as well as a method for adopting and inspection the standards (Gilbert et al., 2015). Certification is based on the idea that it might assist farmers in improving their performance regarding their social life better environment, getting higher pricing, easier market access, and boosting their economic condition. These enhancements are especially essential for small-farmer suppliers in developing nations, who often capture just a tiny portion of the value created in their sector due to their distance from the final customer (Valkila, 2009).

Geographical Indication (GI) is such a certification that refers to a product differentiation procedure that certifies the uniqueness and high quality of the items produced (Teuber, 2007). It is a quality assurance system aimed at preserving local and regional production processes and product integrity (Becker, 2009; Ilbery and Kneafsey, 2000). Galtier, Belletti, and Marescotti (2008) revealed that Geographical Indications can play a different role than product certification programs because the GI standards – including the determination of producing area boundaries and the descriptions of production standards and product quality – is usually elaborated by local actors who can determine the relationship with the terroir (geographic and sociocultural traits). Coffee quality is closely related to agricultural practice and postharvest handling practice technology. In the developing countries, including Indonesia, coffee production is mostly produced by small-scale farmers (Giovannucci and Ponte, 2005) who still apply poor cultivation and postharvest practice resulting in unsatisfactory goods. Minimum standards for coffee, such as geographical indications, require improvements in smallholder farming and postharvest technologies, one of which is achieved through Good Agricultural Practices.

Adopting a certification scheme that promotes quality assurance, such as Geographical Indication, requires a significant amount of work and cost for farmers, especially since farm inspections are required once the scheme is established (Dammert and Mohan, 2014; Giovannucci and Ponte, 2005). Economic motivation, farmers' attitudes toward GI practices, farmers' perceptions of the benefits that will be obtained by applying GI standards, the ease with which GI standards can be implemented, and the extent of farmers' knowledge regarding agricultural practices and good postharvest handling practices could all influence their decision to adopt GI schemes. Several studies have shown the economic effect of GI schemes. Arfini, Cozzi, Mancini, Ferrer-Perez, and Gil (2019) revealed that GI has positive regional and economic impact on the community who live within the production area. GI protection also has an overall beneficial impact on agriculture value added according to Cei et al. (2018). Another study showed that Geographical indication certification provides an economic effect in reducing the cost to build reputation compared to relying solely on trademarks, however GI certification has no influence or negatively affects producers (Menapace and Moschini, 2012). Conversely, according to the findings of empirical recent study in Italy by Cristina (2021) and Santeramo et al. (2021), geographic indication schemes have not given considerable benefits for products or locations of origin. Cristina (2021) reveals that the GIs scheme is still far away from ensuring that all product and place-of-origin benefit from this such scheme due to the regional imbalances and the local economy has not yet fully adapted to the scheme. Meanwhile, Santeramo et al. (2021) shows that, while the GI scheme raises product prices (premium price), but the production and processing costs become higher, so the GI scheme's benefits have not yet been apparent.

Some literature uses non-psychological factors to determine the intention to adopt new technology. Blazy, Carpentier, and Thomas (2011) employed a decision modeling approach to estimate farmers' willingness to adopt agro-ecological advances using non-psychological drivers which are technical nature, economic impact, human capital, and agricultural structure. While Meijer et al. (2014) focused on the impact of farmer knowledge on agricultural and agroforestry innovation uptake in Sub-Saharan Africa. Furthermore, institutional and societal factors were explored by Ngokkuen and Grote (2010) to investigate influencing factors of the adoption of geographical indicator certificates in Thailand. Morello et al. (2018) used drivers such as market access, rural extension, level of education, and experience of farmers to study the influencing factors of farmers' adoption on new ways of using fertilizers. Farmers' socio-demographic and environmental characteristics are used by Sinyolo (2020) to assess their impact on the adoption of enhanced maize varieties.

A review study conducted by Pierpaoli et al. (2013) had discussed the two types of drivers affecting farmers’ adoption in agriculture which is ex-post driver and ex-ante driver. Economic, societal, environmental, and entrepreneurial factors are all ex-post drivers while ex-ante drivers include aspects of human behavior such as perception and attitude. Ex-post research, according to (Pierpaoli et al., 2013), have proven that motivation has encouraged, and likely continue to encourage the adoption of new technologies by farmers, but ex-ante research allowed for investigation the acceptability of a new technology prior to its implementation. According to Useche et al. (2013), these two drivers are useful in interpreting farmer choices when it comes to engaging with new technologies and their adoption in agriculture. However, related to this study, there is still a lack of literature that applied both of these two approaches (ex-post and ex-ante) analysis that affect farmers' adoption of these production-differentiating mechanisms in the coffee industry in this study regarding Geographical Indication. Nonetheless, many studies on the willingness to adopt a technology, scheme, or program that related to coffee certification or other agricultural certification have been conducted, and could be used as references to determine factors that affect farmers’ willingness to accept GI schemes.

This study come up with the current evidence by elucidating the causal linkages between socio-psychological (ex-post and ex-ante) components of attitudes toward behavior (ATB), subjective norm (SN), perceived behavioral control (PBC), perceived economic benefit, perceived usefulness (PU), farmers’ knowledge and socio demographic characteristic in relation to farmers' willingness to adopt geographical indications’ practice and procedures. This model allows for a more comprehensive understanding of small-scale farmers’ decisions, which may be used to improve the performance of Geographical Indication schemes in Indonesia. The aims of this work are (1) to determine the effect of psychological constructs of TPB and TAM on farmers' willingness to adopt GI schemes, (2) to investigate the effect of socio-demographic construct on farmers’ willingness to adopt GI schemes, (3) to investigate relevant causal interrelationships between socio-psychological constructs and their interaction affecting the intention to adopt GI schemes.

## 2 Review of literature

### 2.1 Review of theories

Intention that refers to the motivation or willingness to adopt a certain behavior according to Ajzen’s planned behavior theory, is driven by three constructs which is SN, PBC and ATB, while technology acceptance model approach posits that there are two factors that determine someone willingness to adopt a new technology which is PU and PEOU. Recent research has demonstrated that combining different conceptual models is required to gain a deeper, richer, and more comprehensive knowledge of technology adoption and behavioral intention (Oliveira et al., 2014; Pappa et al., 2018; Williams et al., 2009). TPB and TAM have been widely applied as a theoretical foundation for studies about behavioral intention toward the adoption of technologies (King and He, 2006; Y. Lee, Kozar and Larsen, 2003).

Ajzen's theory of planned behavior (Ajzen, 1991) and Davis' technology acceptance model (Davis, 1989) are two theories that may be used to explain the factors influencing farmers' willingness to accept new technology. Based on these two theories, it is possible to predict peoples’ behavior through their attitude, perception toward such behavior and the influence of external factors such as prominent people around them. These constructs are essential related to such behavior. The relationship between the TPB and TAM constructs and their influence on behavior has been investigated in several previous research topics. Zeweld et al. (2017) had explored TPB and TAM constructs relationship with the intention to implement sustainable agricultural practices in Ethiopia. Jiang et al. (2018) looked at the relationship between the TPB construct and the desire to reuse the waste of agricultural biomass by farmers in China. Moreover, Daxini et al. (2019) examined how the TPB construct affects the farmers intention to use a fertilization management system in China. Furthermore, Castillo et al. (2021) combined the TPB construct and social capital factors to study farmers behavior in adopting irrigation technology. In the coffee sector, Nguyen and Drakou (2021) used the TPB constructs to study coffee farmer’s behavior to adopt a sustainable agricultural system that responds to climate change in China. The TPB construct was also developed to gain deeper knowledge related to human behavior, as was conducted by Wang and Scrimgeour (2021) who combined the TPB construct with motivation theory and attachment theory to understand consumers behavior in adopting plant-based diet in China.

According to Ajzen (2005), attitudes toward behavior (ATB) can be driven by the belief in the effect of a behavior or can be referred to as behavioral beliefs. It can be something that is favorable or unfavorable, depending on their appraisement. For instance, if farmers think that the certification program could provide benefits for them, then they would give a positive response to it. Conversely, if they think that the certification program did not provide benefits for them, they would give a negative response. This theory also explains the variables that could be associated with or influencing the beliefs held by individuals such as: age, gender, socioeconomic status, education, group membership, past experience, social support, and etc. In terms of performing behavior, Armitage and Conner (2001) stated that individual preferences are reflected by attitude and the attitude of an individual may reveal his or her intention to express behavior. The higher the willingness to exhibit a certain behavior, then they'll look for a more favorable reaction to the behavior. Perceived behavior control (PBC), is the perceived difficulty or easiness in conducting certain behaviors by someone that is influenced by their assurance on the easiness to access resources such as equipment, compatibility, competence, and opportunity (Icek Ajzen, 2005). It also relates to the individual's perspective of the conditions which might enhance or inhibit a behavior's expression (Guido et al., 2010). Subjective norms (SN) refer to Ajzen’s TPB explained as “*perceived social pressure to perform* or not to perform the behavior” (Icek Ajzen, 1991). People are more likely to adhere to subjective norms since they are concerned of social exclusion (Bamberg and Möser, 2007). As a result, if farmers believe in the opinion of other people, they value agrees with them on a certain behavior, they would be more likely to indulge in that particular behavior (Rezaei et al., 2018).

Similar to ATB and PBC as described in Ajzens’ planned behavior theory, perceived ease of use (PEOU) and perceived usefulness (PU) as proposed in Technology Acceptance Model (TAM), are also the aspect that affecting peoples’ intention for adoption (Venkatesh and Davis, 2000). According to Davis (1989), PU is a belief or experience of person that the introduced technology being used can improve their performance, whereas PEOU refers to a person's perception that adopting new technologies is not onerous. Brosnan (1999) stated that PU and PEOU can have a direct impact on willingness to adopt a certain behavior. Davis (1989) argued that PU had a stronger impact than that of PEOU. External factors can also be mediated through PU and PEOU, resulting in an indirect effect on the willingness to adopt technology (Venkatesh et al., 2003). While referring to Pan et al. (2005), PEOU may be considered as both an external and an internal variable influencing the willingness to accept a certain behavior. PEOU is also considered as the most significant construct in the theory of technology acceptance model and has been empirically suggested as a major driver of adoption intention (Agarwal and Karahanna, 2000; Amin et al., 2014).

### 2.2 Review of empirical studies

#### 2.2.1 The effect of TPB and TAM construct on intention

Prior studies have proven that the effect of the constructs of TPB on behavioral intention and the inter-relationship between the constructs of TPB can vary. Daxini et al. (2019) found that the three cores of TPB constructs have shown a positive effect on farmers’ intention to accept riparian land management. Another study has proven the same result, where attitude toward behavior, subjective norms and perceived behavioral control had affected positively the farmers’ willingness to accept sustainable agricultural practice (Zeweld et al., 2017), Consumers' intention to consume a plant-based diet (Wang and Scrimgeour, 2021), prediction of organic food consumption (Scalco et al., 2017), engaging in managing food safety (Rezaei et al., 2018) and intention to adopt greywater treatment technologies (Oteng-Peprah et al., 2019). However, some previous studies have shown different results regarding the effect of TPB construct on behavior intention, it could vary depending on the field of study. Jiang et al. (2018) revealed that the two constructs of TPB, PBC and ATB significantly affect farmers’ willingness in reusing agricultural biomass waste, but nor for subjective norms. Pappa, Iliopoulos, and Massouras (2018) only found a single construct of TPB which is PBC that significantly and positively affects farmers’ willingness to use electronic traceability applications. Eweoya et al. (2021) found that the only one of TPB constructs which is ATB that significantly affects farmers’ intention to adopt soil management practice in Belgium. Prior studies also have shown that there are interrelationships among the three constructs of TPB that influence behavioral intention (Bamberg et al., 2007; Borges et al., 2016; Jiang et al., 2018; Morais et al., 2018; Oteng-Peprah et al., 2019; Quintal et al., 2010; Scalco et al., 2017; Wang and Scrimgeour, 2021).

Furthermore, in terms of the construct of TAM, PEOU has no effect on intention behavior. According to Halilovic and Cicic (2015), it depends on the subject area of study. For instance, the adoption of smart farming technologies did not show a direct effect between PEOU and behavior intention (BI), while PU did (Caffaro et al., 2020). Another study about farmers’ participation in vegetable traceability systems, conducted by Li, Paudel, and Guo (2021), has shown that there is no a significant relationship between PEOU and Intention, the finding also revealed that there was no significant effect between PEOU and PU. Furthermore, PEOU that was reinterpreted into perceived cost in a study of adopting alley cropping system in German showed that there is no significant effect of perceived cost on behavioral intention (Otter and Beer, 2021). Several studies have shown significant influence of PEU on behavior intention in different subject area of research, such as adoption of e-agriculture in Nigeria (Eweoya et al., 2021); adoption of smart meters in Taiwan, Korea and Indonesia (Chou et al., 2015); and user contentment using mobile website (Amin et al., 2014). PEOU – refers to the unified theory of acceptance and use of technology (UTAUT) – is known as effort expectancy. While perceived usefulness in previous study was also defined as perceived value and benefit (L. Li et al., 2021). In term of GIs’ coffee schemes, the perceived benefit of GI for farmers is to obtain knowledge that can explain their willingness or intention to adopt GIPs’ practice and procedures, and the main purpose of geographical indication is also to seize financial benefits associated with GIs’ uniquely standards (place and process) (Neilson et al., 2018).

#### 2.2.2 The effect of socio-demographic characteristics of farmers on TPB and TAM constructs

Previous studies have considered another determinant factor that influences behavioral intention which is knowledge as a manifest variable that represents farmer understanding of such behavior to be adopted. According to Xu and Zhang (2021) the relationship between knowledge, attitude and behavior usage can be described as the following framework where knowledge is the basic understanding of concept, attitude is the motivation and behavior usage is the goal. Prior study such as Govindharaj et al. (2021) has shown that the variable of knowledge significantly affects the variable of SN, PBC, and behavioral intention. In some cases, some studies have shown that knowledge can determine farmers’ attitude and influence behavior indirectly (Cao et al., 2009; Meijer et al., 2014; Tran et al., 2018). Bamberg and Möser (2007) revealed that knowledge has direct influences on either perceived behavior control or attitude toward behavior, asserted by Icek Ajzen (1991), stated that people decision to choose such behavior do not entirely depend on their subjective intention, but their choice limited by specific objective such as knowledge, resource and opportunity. Previous studies have shown that the adoption of new agricultural technology or systems can be influenced by farmers’ knowledge. A study conducted by Nyang'au et al. (2021) shows that knowledge is one of the determinants that influence the adoption of climate smart agricultural practice in Kenya along with other factors farmers’ perception and attitude. Other studies (Bagheri et al., 2019; Govindharaj et al., 2021; Tama et al., 2021; Tran et al., 2018) had shown that knowledge significantly affects behavioral intention.

Considering the socio-demographic characteristics of famers are important to give more understanding and knowledge about its influences on individual behavior to a specific subject. Senger et al. (2017) stated that famer’s socio-demographics can be considered as background variable explaining their effect on intention. Previous study conducted by Rezaei et al. (2019) has considered socio-demographic characteristics into their developed model. According to Nyang'au et al. (2021), farmers' adoption decisions can be explained by socio-demographic characteristics of farmers: household size, income and credit access. Tran et al. (2018) highlighted that age, level of education, occupation, level of income and access to communication can influence farmers’ perception to adopt land use and cover changes management. Pierpaoli et al. (2013) summarizes from several prior studies, shows that farmers’ socio-demographic factors (age, experience, education level, self-confidence, and social factors) significantly affect the individual’s attitude to adopt technology. A study conducted by Saengavut and Jirasatthumb (2021) about technology adoption intention in Thailand has shown that socio-demographics characteristics of farmers give a significant and positive effect on the willingness to adopt technology. Zhou et al. (2019) has proven that socio-demographics significantly affect the human behavior. However, another study shown different result, such as Fernandes et al. (2021) conducted a study in Indonesia about farmers satisfaction has shown that socio-demographics variable did not significantly influence farmers’ gratitude.

## 3 Conceptual framework and hypotheses

Intention that refers to the motivation or willingness to adopt a certain behavior according to Ajzen’s planned behavior theory, is driven by three constructs which is SN, PBC and ATB, while technology acceptance model approach posits that there are two factors that determine someone willingness to adopt a new technology which is PU and PEOU. Recent research has demonstrated that combining different conceptual models is required to gain a deeper, richer, and more comprehensive knowledge of technology adoption and behavioral intention (Oliveira et al., 2014; Pappa et al., 2018; Williams et al., 2009). TPB and TAM have been widely applied as a theoretical foundation for studies about behavioral intention toward the adoption of technologies (King and He, 2006; Y. Lee, Kozar and Larsen, 2003). The constructs of TPB and TAM have been used and developed in various ways and different subject areas. Several studies have combined the constructs of both models to develop an adaptable framework for varied technological and organizational contexts. Chen (2016) conducted a study that revealed that PEOU and SN have the greatest impact on public bike system loyalty. M.-C. Lee (2009) showed that perceived benefit, ATB and PU influenced the willingness to use internet banking. Furthermore, Salo et al. (2021) revealed that SN and PEU were the main constructs of TPB and TAM in influencing small farmers in Colombia to adopt insect rearing activity for animals. Another research by Akyüz et al. (2020) from behavioral studies in Turkey on willingness to apply and sustain organic farming practices, demonstrated that the subjective norm is a major driver for organic agriculture adoption and maintenance. While Mohr and Kühl (2021) found that perceived behavioral control (PBC) was the strongest predictor among two constructs, TAM and TPB, in influencing farmers in Germany to accept artificial intelligence in agriculture.

**Figure 1:**
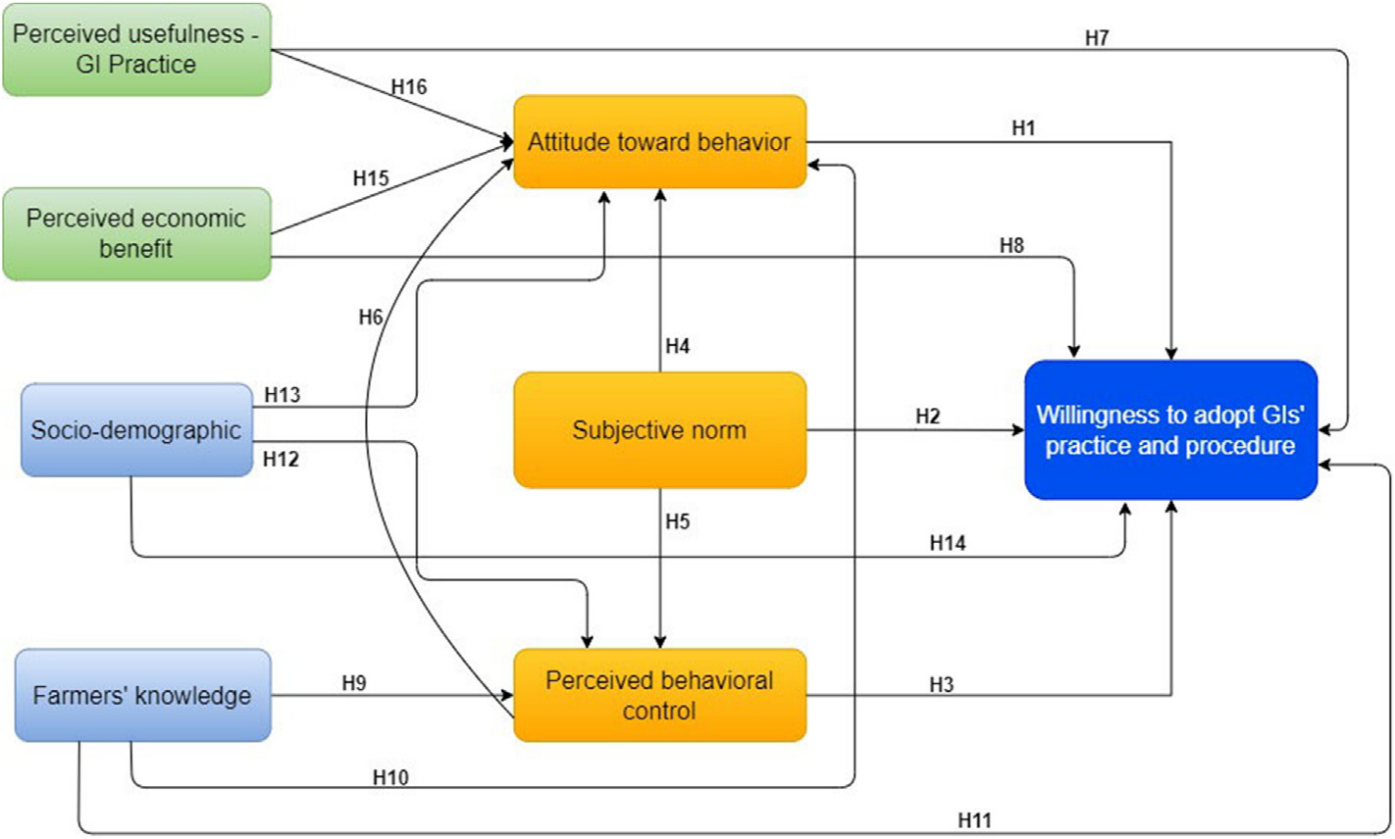
Proposed theoretical model.

Our research model tries to combine some core constructs of TPB and TAM for a more suitable framework in our field study. We argued that by combining these two theories could give more understanding of Geographical Indication scheme adoption in the coffee sector in Indonesia, to the point where our proposed framework is applicable and referring to specific variables that can affect farmers willingness to adopt GIs’ schemes. The basic assumption of the model proposed in this study is that willingness to adopt Geographical Indication practice and procedures is influenced by (1) Subjective Norms (SN) which are expressed through perceived 'external pressure' in the form of suggestions or invitation from influential stakeholders such as extension officer, farmers’ group and community of GI protection (MPIG) in adopting GI schemes, (2) Attitude Toward Behavior (ATB), which is formed by the perception of the standard (code of practice) of the GI’s scheme and (3) Perceived Behavioral Control (PBC) which is constructed by the farmer's perception of the easiness of the implementation of GI’s standard (code of practice). Figure 1, which represents our suggested theoretical model, depicts relationships among variables and shows the hypotheses to be investigated. The structural equation, consisting of the interaction among variables, while their indicators are not depicted in detail, in order to be more focused on our proposed model. All variables and their specific indicators were described and explained in Table 2.

In our developed model, Subjective Norms (SN) is defined as farmers’ response to important referents such as extension officers, MPIG leadership or staff, and head of farmers to adopt GIP. We argue that SN has a direct effect on farmers’ willingness to implement geographical indication practice that can be explained that people typically choose to do such behavior because they are influenced and motivated by important referents around them (Schepers and Wetzels, 2007). The more positive SN, the more likely the behavior will be adopted (Icek Ajzen, 1991). We also predict that SN would directly affect ATB and PBC. In terms of the construct of behavioral intention based on TPB, in this study, we focus only on the relationship and the direction effect of SN on ATB and PBC. We argue that SN could affect farmers’ attitude toward the adoption of GIs’ standards due to farmers' access to information through significant referent groups that can be utilized to validate the benefits they will obtain if they adopt GI (Bamberg and Möser, 2007). While the perceived behavior control (PBC) could be affected by subjective norms in terms of individual perceptions of the easiness or difficulties in performing a certain behavior (Quintal et al., 2010).

Furthermore, ATB in this study is described as how an individual evaluates (favorable or unfavorable) the GIs’ code of practice, which is in line with Ajzen’s TPB (Icek Ajzen, 1991). Farmers' willingness to adopt Geographical Indications (WTA-IG) schemes will thus increase if farmers believe that implementing GIs’ standards is useful and advantageous to them and will result in positive outcomes. Furthermore, farmers’ attitude toward GIP is reflected by PU and PEB. Refers to Davis’s TAM theory (1989), PU is defined as the degree to which an individual feel that implementing a specific new system will enhance the work performance. PU in this study is divided into variables which are Perceived Usefulness of Geographical Indication Practice (PU-GIP) and Perceived Economic Benefit of Geographical Indication schemes (PEB). PU-GIP is defined as farmers’ perception of the advantages implementing geographical indication practice and procedures, particularly in the execution of GIs’ code of practice (production system), perceived by farmers. While perceived economic benefit (PEB) is defined as farmers' perceptions of the economic benefits that will be obtained from the selling price of coffee produced based on geographical indication standards. PEB is also considered as a relative phrase that indicates one's outlook on the future regarding the economic benefit of the performance of geographical indication practices, which is similar to financial contentment (Gasiorowska, 2014), and a perception from one's economic status in comparison to others (Karraker, 2014). Both of the variables, perceived of usefulness and perceived of economic benefit (PEB) are predicted to have a significant effect on ‘Attitude toward behavior’ to adopt GI. We hypothesize that there is a direct effect from PU-GIP and PEB to WTA-IG rather than an indirect effect. We argue that these variables (PU-GIP and PEB) have direct influence on farmers’ willingness to adopt GI schemes, as stated by Venkatesh and Davis (1996) – refer to the final version of technology acceptance model – revealed that PU and PEB could affect behavioral intention directly. Supporting our argument, we refer to previous study that found PU had demonstrated as a powerful predictor in predicting behavioral intention rather than ATB, according to Yang and Yoo (2004). Another argument argued that the primary motivation for implementing a new technology is to increase profitability (Pierpaoli et al., 2013).

In addition, PBC is explained as the farmers’ perception of the geographical indication code of practice and what they believe in the ease or difficulty of performing geographical indication code of practice. This variable is related to farmer’s current condition and situation of farmers that could be affected by the constraints and ease faced by farmers (I. Ajzen, 2002; Bamberg and Möser, 2007; Gong and Yan, 2004). PBC also refers to an individual's perspective of the aspects that may encourage or inhibit a behavior's expression (Guido et al., 2010). How strong the PBC in affecting behavioral intention could be varies across studies. Several studies had found that PBC was significant in influencing intention (Ataei et al., 2021; Govindharaj et al., 2021; Krishnadas and Renganathan, 2021; Oteng-Peprah et al., 2019; Savari and Gharechaee, 2020; Scalco et al., 2017). However, other studies found that PBC insignificant affecting intention (Tama et al., 2021; Wauters et al., 2010). We argue that farmers’ PBC over the adoption of Geographical Indication code of conduct is determined by farmers’ PEOU of implementing GIs’ code of practices. Therefore, in our model, PBC has a direct effect on willingness to adopt GIs’ schemes. The PEOU by Farmers in performing geographical indication code of practice is predicted to significantly affect attitudes toward adopting GI’s code of practices.

We considered knowledge in our model as recently studies have shown that knowledge can influence attitude on behavior (Govindharaj et al., 2021), such as in diffusion of innovation process (Taherdoost, 2018). This variable has been studied extensively since it is widely acknowledged that the better of an individual’s understanding of particular behavior would affect a more influential attitude toward a certain behavior (Bagheri et al., 2019). Many recently studies had put into account knowledge to acquire more information about the effect of individual knowledge on behavioral intention, ATB, SN and PBC (Bagheri et al., 2019; Govindharaj et al., 2021; Tama et al., 2021; Tran et al., 2018). In our study, knowledge is explained as the understanding about the geographical indication code of practice in terms of (1) Good Agricultural Practice of Coffee and (2) postharvest standard. Inspired by the previous studies (Bagheri et al., 2019; Govindharaj et al., 2021; Tama et al., 2021) using extended Ajzen’s TPB considering knowledge as one of the variables influencing the behavioral intention, ATB, SN, and PBC, our research will be focused on the impact of farmers’ knowledge on the core element of TPB and TAM exclude subjective norm. We test the hypothesis that farmers' willingness to adopt GI, their perception of behavioral control, and their attitude toward behavior would all improve as their understanding and knowledge of the GI code of practice improves.

Finally, although this study focuses on the core elements of TPB and TAM constructs associate with farmers willingness to adopt GIP, we take into account socio-demographics characteristics of farmers in our model to give more information related to farmers characteristics background that can be considered as a variable that would influence farmers’ decision to adopt GIs’ schemes. In our model the socio-demographics variables are represented by age, experience and managed farm size area. Inspired by several studies ((Fernandes et al., 2021; W. Li, Wei, Zhu and Guo, 2019; Zhou et al., 2019), we considered sociodemographic variable in this study as a exogenous latent variable that expected has significant effect on attitude toward adopting GI’s standards, perception of behavioral control and willingness to adopt GIs’ standards.

We came up with the following hypotheses based on our theoretical model:

H1 Willingness to adopt GIP Is positively affected by attitude.

H2 Willingness to adopt GIP is influenced by subjective norm.

H3 Willingness to adopt GIP is positively affected by perceived behavioral control.

H4 Attitude is positively affected by subjective norms.

H5 Perceived behavioral control is positively affected by subjective norms.

H6 Attitude is positively affected by perceived behavioral control.

H7 Willingness to adopt GIP is positively affected by perceived usefulness.

H8 Willingness to adopt GIP is positively affected by perceived economic benefit.

H9 Perceived behavioral control is positively affected by farmers’ knowledge.

H10 Attitude is positively affected by farmers’ knowledge.

H11 Willingness to adopt GIP is positively affected by farmers’ knowledge.

H12 Perceived behavioral control is positively affected by socio-demographic.

H13 Attitude is positively affected by socio-demographic.

H14 Willingness to adopt GIP is positively affected by socio-demographic.

H15 Attitude is positively affected by perceived usefulness of GIs’ practices.

H16 Attitude toward behavior is positively affected by perceived economic benefit.

## 4 Material and methods

### 4.1 Study area

The research was carried out in two different coffee producing regions that have been awarded GI certification. First location is arabica coffee producing region of Sindoro-Sumbing located around Mount Sindoro and Mount Sumbing with an altitude above 900 m to 2,100 m above sea level. Sindoro and Sumbing highlands administratively located in three regencies in central java province, that is Wonosobo, Temanggung and Magelang. The second research location is the GI protected area of Temanggung Robusta coffee. The GI protected area of temanggung robusta coffee is situated in the lowland of Temanggung, Central Java. Robusta coffee plantations in Temanggung situated from midland to highland area with an altitude from 400 m–1200 m above sea level with topographical characteristics varying from flat to hilly. The highland of Temanggung also known as one of central production areas of tobacco in Indonesia where arabica coffee plants are grown as conservation plants and intercropping plants with tobacco and horticulture crops, while others are grown in the protected forest of Mount Sindoro and mount Sumbing.

### 4.2 Sample and data collection

Research respondents were distributed in two GIs' protected areas in Temanggung regency's primary coffee growing areas. The data was gathered by a face-to-face interview with respondents using questionnaire survey in six districts that represented the main production areas of Robusta and Arabica coffee in Temanggung, namely Gemawang, Kandangan, Candiroto, Bejen, Ngadirejo and Kledung districts. Informed consent was obtained from all respondents who participated in this survey. The survey was done from November 2020 to March 2021. Respondents that took part in this study were chosen using a purposive sample technique and were selected based on several criteria including: 1) respondents are coffee farmers who process coffee into green bean coffee; and 2) the respondent has received socialization or information about geographical indication certification requirements. Therefore, because the population of coffee farmers who process coffee cherries into green beans is not available, the option of using random sampling is not possible. A total of 178 respondents consisting of 90 respondents of robusta coffee farmers and 90 respondents of arabica coffee farmers with two outliers being the arabica coffee respondents. The number of respondents in our research has fulfilled the minimum requirement of PLS-SEM application. PLS-SEM requires at least a sample size of ten times the number of arrowheads pointing at our latent variable (16 directions) which is equal to 160 (J. Hair, Hult, Ringle and Sarstedt, 2014).

The materials of the survey were designed on the basis of a literature study of prior TPB and TAM research in agricultural sector (Caffaro et al., 2020; Daxini et al., 2019; Jiang et al., 2018; Pappa et al., 2018; Verma and Sinha, 2018; Zeweld et al., 2017). The interviews were purposed to identify important attitudes and perspectives for adopting GIP. The questionnaire used in this study is a self-designed questionnaire. A structured two-part questionnaire was used in this survey. The first section consisted of socio-demographic characteristics of respondents. The other section contained a set of questionnaires to evaluate farmers' construction characteristics of TPB, TAM, knowledge and socio-demography, including 33 indicators from eight subsection: (1) six WTA indicators, (2) four ATB indicators, (3) five PBC indicators, (4) four SN indicators, (5) four PU-GI indicators, (6) three PU-EB indicators, (7) four respondents' knowledge indicators, and (8) three socio-demographic indicators (Table 1). Each item played the role as reflector of the respective construct of TAM, TPB, Knowledge, and Socio-Demographic. A 1–5 likert scale was implemented to rate farmers’ responses (exclude socio-demographic items) ranging from strongly disagree to absolutely agree. A detailed description of the research variables was depicted in Table 2. The respondent sociodemographic variables are included such as age, experience, and managed farm size area.

**Table 1:** Socio-demographic characteristics of coffee farmers.

Variable	Category	Frequency	Percent
Age (year)	<26	8	4.49
	26–35	31	17.42
	36–59	123	69.10
	60–65	7	3.93
	>65	9	5.06
Education	Illiterate	0	0
	Elementary	61	34.27
	Secondary	42	23.60
	High school	62	34.83
	College education	13	7.30
Experience (year)	<10	102	57.30
	10–20	41	23.03
	21–30	26	14.61
	31–40	7	3.93
	>40	2	1.12
Under cultivation of coffee (ha)	<0.25	17	9.55
	0.25 to <0.5	50	28.09
	0.5 to <1	57	32.02
	1 to 2	44	24.72
	>2	10	5.62

**Table 2:** Research measurement, variables and standardized factor loading.

Construct	Measurement items	factor loading
Willingness to Adopt (WTA)	• I tend to keep farm records for GI verification (WTA1).	0.653^a^
	• I tend to selectively pick red cherries (WTA2).	0.720^a^
	• I will process coffee beans according to GIs’ code of practice (WTA3).	0.719^a^
	• I am willing to process green beans based on GI recommendation (**Robusta**: full washed, natural, honey; **Arabica:** full washed) (WTA4).	0.666^a^
	• I am willing to clean and sort the harvested cherries by floating the cherries in the water (WTA5).	0.817^a^
	• I tend to use only good cherries to be processed (WTA6)	0.768^a^
Attitude Toward Behavior (ATB)	• I believe keeping farm records will help me manage my farm (ATB1).	0.743^a^
	• I believe the implementation of GIs’ code of practice will increase my income (ATB2).	0.762^a^
	• I believe picking only red cherries is the right way to harvest coffee (ATB3).	0.737^a^
	• I believe sorting and cleaning by floating the coffee cherries in the water will improve the quality of green beans (ATB4).	0.724^a^
Perceived Usefulness of Geographical Indication Practice (PU-GI)	• The GIs’ practice and procedure will give some benefit (PU-GIP1)	0.843^a^
	• Participating in community of GI protection will give me several advantages (PU-GIP2)	0.775^a^
	• The GIs’ code of practice will improve the quality of coffee produced (PU-GIP3).	0.767^a^
	• The GIs’ code of practice will increase the profit from higher selling price of green beans (PUG-GIP4).	0.549^a^
Perceived Economic Benefit (PEB)	• The price of coffee that is processed based on GI standards is higher than the price of non-GI coffee (PEB1).	0.866^a^
	• Coffee processors and buyers offer higher prices for coffee processed under GI standards (PEB2).	0.787^a^
	• Farmers have obtained economic benefits through the GIs' code of practice (PEB3).	0.903^a^
Perceived Behavioral Control (PBC)	• GI-based coffee production requires coffee cherries to be floated in the water for sorting and cleaning (PBC1)	0.851^a^
	• The GI standard requires that arabica green bean is processed by full washed method, while Robusta is processed by full washed, dry processed and honey method (PBC2).	0.694^a^
	• I believe I can selectively pick only red cherries (PBC3).	0.523^a^
	• I believe I can do sorting and cleaning by floating coffee cherries in the water (PBC4)	0.871^a^
	• I believe I can process my green beans and meet the GIs’ code of practice (PBC5).	0.811^a^
Subjective Norm (SN)	• MPIG management/head of farmers' group/extension officers suggested that I adopt GIs' code of practice (SN1).	0.780^a^
	• MPIG management/head of farmers’ group/extension officers suggested that I do selective harvesting (manually), only picking red coffee cherries (SN2).	0.832^a^
	• MPIG administrator/farmer group leader/extension worker, advised me to sort and clean the coffee cherries by floating the cherries in the water (SN3).	0.859^a^
	• MPIG management/head of farmer group/extension officers, suggested that I process green beans based on IG standards (SN4).	0.718^a^
Farmers’ Knowledge (FK)	• I know that green beans processing based on GI standards only use red cherries (FK1)	0.749^a^
	• I know that coffee sorting and cleaning is done by floating the coffee cherries in the water (FK2).	0.844^a^
	• I know that the floating cherries must be separated during coffee processing (FK3)	0.792^a^
	• I know that based on GI code of practice, damaged, unripe, and overripe cherries are not allowed to be used in green beans processing (Arabica: full wash; Robusta: full wash, dry process, honey) (FK4)	0.710^a^
Socio-Demographic (SD)	• Age (years) (1) < 26; (2) 26–35; (3) 36–59; (4) 60–65; (5) > 65	0.783^a^
	• Experience (years)→ farmers’ coffee farming experience. (1) < 10; (2) 10–20; (3) 21–30; (4) 31–40; (5) >40	0.820^a^
	• farm size area (hectare)→ the farm size area that managed by farmers for coffee plantation. (1) < 0.25; (2) 0.25 - < 0.5; (3) 0.5 - < 1; (4) 1–2; (5) > 2	0.679^a^

a significant at P < 0.001.

### 4.3 Data analysis

Partial least square (PLS) analysis was employed in this study to estimate the study model and its predictive value as a component of structural equation modeling (SEM). PLS modeling is a variance-based prediction method focusing on endogenous constructs in a model to maximize the variance explanations (Hair et al., 2014), which is suitable for exploratory research objectives (J. Hair, Ringle and Sarstedt, 2012). PLS-SEM according to J. Hair, Sarstedt, Ringle, and Mena (2012) is attractive to deal with small sample size problems and the data is not normal. Therefore, PLS-SEM is a suitable alternative for covariance-based SEM that allows for the construction and estimation of a particular model without considering high restricting constraints (J. F. Hair, Sarstedt, Pieper and Ringle, 2012; J. Hair, M. Sarstedt, et al., 2012). PLS-SEM and confirmatory factor analysis (CFA) with WarpPLS software version 7.0 were adopted in analyzing the data and address the hypotheses of the study. Two steps of measurement will be taken into account to answer our hypotheses. First, the assessment of the outer model including the measurement of reliability of indicator, convergent validity, internal consistency and discriminant validity. Secondly, the overall fit of the model was measured by using the tenenhaus GoF criterion (Tenenhaus et al., 2004).

The SEM model is represented in Figure 1, Farmers' willingness to adopt GI schemes is treated as an endogenous latent variable in the hypothesis model and perceived usefulness, perceived economic benefit, attitude toward behavior, subjective norm, perceived behavioral control, socio-demographics and farmers' knowledge as exogenous latent variables. Multi-item scales were used to measure the latent variable. The SEM comprises two models: measurement and the structural models. The relationship between observable and latent variables is described by the measurement model. The endogenous and exogenous latent variables are linked in the structural model. The structural equation model consists of three matrix equations, as follows:

(1) β=Aβ+Bλ+ζ

(2) X=Λxλ+v

(3) Y=Λyβ+ε

Eq. (1) is the structural model, where β represents the endogenous latent variable, λ refers to the exogenous latent variable, both of endogenous and exogenous latent variable linked through the coefficient matrices A and B, and ζ is the error vector. The measurement models are shown in Eqs. (2) and (3). X refers to the observed exogenous latent variable and Y refers to the observation variable of the endogenous latent variable; Λx indicates the matrix of correlation coefficient between the exogenous and its observed variable; Λy indicates the matrix of correlation coefficient between the endogenous and its observed variable.

## 5 Result and discussion

### 5.1 Socio-demographic characteristics

Table 1 shows that the respondents in this survey were overwhelmingly between the ages of 36 and 59 years old (69.1 %). Millennial farmers, defined as those under the age of 26, account for 4.49 percent of responses. The majority of respondents have completed high school (34.83 %), followed by respondents with an education level of elementary school (34.27%). Some of the respondents have attained a university diploma (7.3 %). Coffee farmers with farming experience less than 10 years made up the majority of respondents (57.3%), with an average level of experience of 13 years. Furthermore, based on the farmland area under cultivation of coffee, both owned and rented by farmers, most of the farmers in this research own land between 0.5 – 1 ha (32.02%). Where the average land area of Robusta coffee farmers is larger than that of Arabica coffee farmers, 1.11 ha compared to 0.44 ha. Coffee farming is the primary source of livelihood for mostly robusta coffee farmers. On the contrary, the majority of respondents of arabica coffee farmers in this study put coffee farming as a secondary source of their livelihood after tobacco and horticulture.

Sindoro-Sumbing Arabica and Temanggung Robusta Coffee are two coffee products from Temanggung Regency that have received GI certificates. Sindoro-Sumbing Java Arabica Coffee received GI certification in 2014, cultivated on the slopes of Mount Sindoro and Mount Sumbing in Central Java at an elevation of over 1000 m above sea level intercropped with tobacco and vegetables. Arabica coffee was also grown as a conservation plant in Temanggung Regency. Temanggung Robusta Coffee received GI certification in 2016 in the lowlands, grown at elevations ranging from 500 to 842 m above sea level. According to the Indonesian National Statistics Agency (2022), the production of Arabica coffee in Temanggung Regency reached 876.19 tons in 2021. Meanwhile, Temanggung Robusta coffee production reached 10,434.48 tons. Total production in Temanggung Regency accounts for around 56% of total coffee production in Central Java Province. Temanggung coffee exports in 2019 reached 15,000 tons (BPS, 2022), while Indonesia's coffee export volume in the same year reached 359,000 tons (BPS, 2021). Egypt, South Korea, and India are among Temanggung's coffee export destinations. In Indonesia, coffee consumption has risen steadily during the last five years, rising from 285,000 tons in 2017 to 300,000 tons in 2021 (ICO, 2021).

### 5.2 Models of measurement and evaluation

The good fit of our model was evaluated using the analysis of the confirmatory factor. As presented in Table 2, all of the estimated loading of measured items was above the recommended level (>0.5) and significant at P < 0.001, where the minimum factor loading was 0.523. According to most references, especially J. Hair, Black, Babin, and Anderson (2010), to be deemed as a strong enough validation for justifying the latent variable, the loading factor's value number should be 0.50 or higher. The values of Average Variance Extracted (AVE), Composite Reliability (CR) and Cronbach's alpha (CA) are presented in Table 3, while the discriminant validity analysis is shown in Table 4. The study reveals that all AVE values – the ability to measure the variation of each latent variable for every measurement subject – were considered acceptable as all the values were above 0.5, ranging from 0.527 to 0.728. Furthermore, the value of CR that indicates the problem’s internal consistency, for all variables was above 0.7 which has met the minimum recommended level (>0.7). The value of CA for all latent variables was above 0.7 except socio-demographic (0.639). Mostly, previous study recommend CA coefficient must be greater than 0.7, however some studies have accepted the level of CA coefficient greater than 0.6 (Borges et al., 2014; Bruijnis et al., 2013; Senger et al., 2017; Tama et al., 2021).

**Table 3:** Results of reliability and convergent validity analysis.

	PU-GIP	PEB	ATB	PBC	WTA	FK	SD	SN
Composite reliability	0.827	0.889	0.830	0.870	0.869	0.857	0.806	0.876
Cronbach's alpha	0.719	0.811	0.727	0.809	0.819	0.777	0.639	0.810
Average Variance Extracted	0.550	0.728	0.550	0.579	0.527	0.601	0.583	0.639

**Table 4:** Results of discriminant validity analysis.

	PU-GIP	PEB	ATB	PBC	WTA	FK	SD	SN
PU-GIP	0.742							
PEB	0.13	0.853						
ATB	0.342	-0.018	0.742					
PBC	0.271	0.119	0.573	0.761				
WTA	0.283	0.209	0.512	0.58	0.726			
FK	0.131	0.342	0.3	0.409	0.497	0.775		
SD	0.004	-0.033	-0.263	-0.214	-0.225	-0.145	0.763	
SN	0.214	-0.063	0.344	0.139	0.175	0.044	0.103	0.799

According to George and Mallery (2016), it is acceptable to have a CA coefficient greater than 0.6. The coefficient of CA greater than 6 shows that the different beliefs or statements can be used to measure willingness to adopt, ATB, PBC, SN, PU, PEB, farmers’ knowledge and socio demographic characteristics of farmers (Borges et al., 2014). Finally, we conducted the discriminant validity analysis to ensure there is no significant correlation between the measurement indexes of different latent variables. The AVE’s value of each construct in the model (Table 4) is higher than the squared value of the correlation coefficient of each latent variable, in this way hinting that our suggested model has good discriminant validity (W. Li et al., 2019).

The fitness of our proposed model was verified using model fitness and quality indices. All variables proposed in our research model confirm the goodness of fit (Table 5). Model fit and quality indices used in this study include average block VIF (AVIF), average full collinearity VIF (AFVIF), sympson’s paradox ratio (SSR), R-squared contribution ratio (RSCR), Statistical suppression ratio (SSR), nonlinear bivariate causality direction ratio (NLBCDR), average path coefficient (APC), average R-squared (ARS), average adjusted r-squared (AARS) and tenenhaus GoF (GoF). The GoF index was applied to evaluate the fit of the overall model. Since the reported value of the GOF index in this model is 0.5, and the lowest allowed level is 0.36, then the research model could be seen to be stable and well-fit.

**Table 5:** Model fit and quality indices.

Model fit index	Evaluation standard	Actual value
Average block VIF (AVIF)	≤3.3 (Ideally)	1.253
Average full collinearity VIF (AFVIF)	≤3.3 (Ideally)	1.497
Tenenhaus GoF (GoF)	small ≥0.1, medium ≥0.25, large ≥0.36	0.5
Sympson's paradox ratio (SPR)	acceptable if ≥ 0.7, ideally = 1	0.938
R-squared contribution ratio (RSCR)	acceptable if ≥ 0.9, ideally = 1	0.996
Statistical suppression ratio (SSR)	acceptable if ≥ 0.7	1
Nonlinear bivariate causality direction ratio (NLBCDR)	acceptable if ≥ 0.7	0.969
Average path coefficient (APC)		0.193∗∗∗
Average R-squared (ARS)		0.421∗∗∗
Average Adjusted R-squared (AARS)		0.404∗∗∗

### 5.3 Hypotheses testing

**Figure 2:**
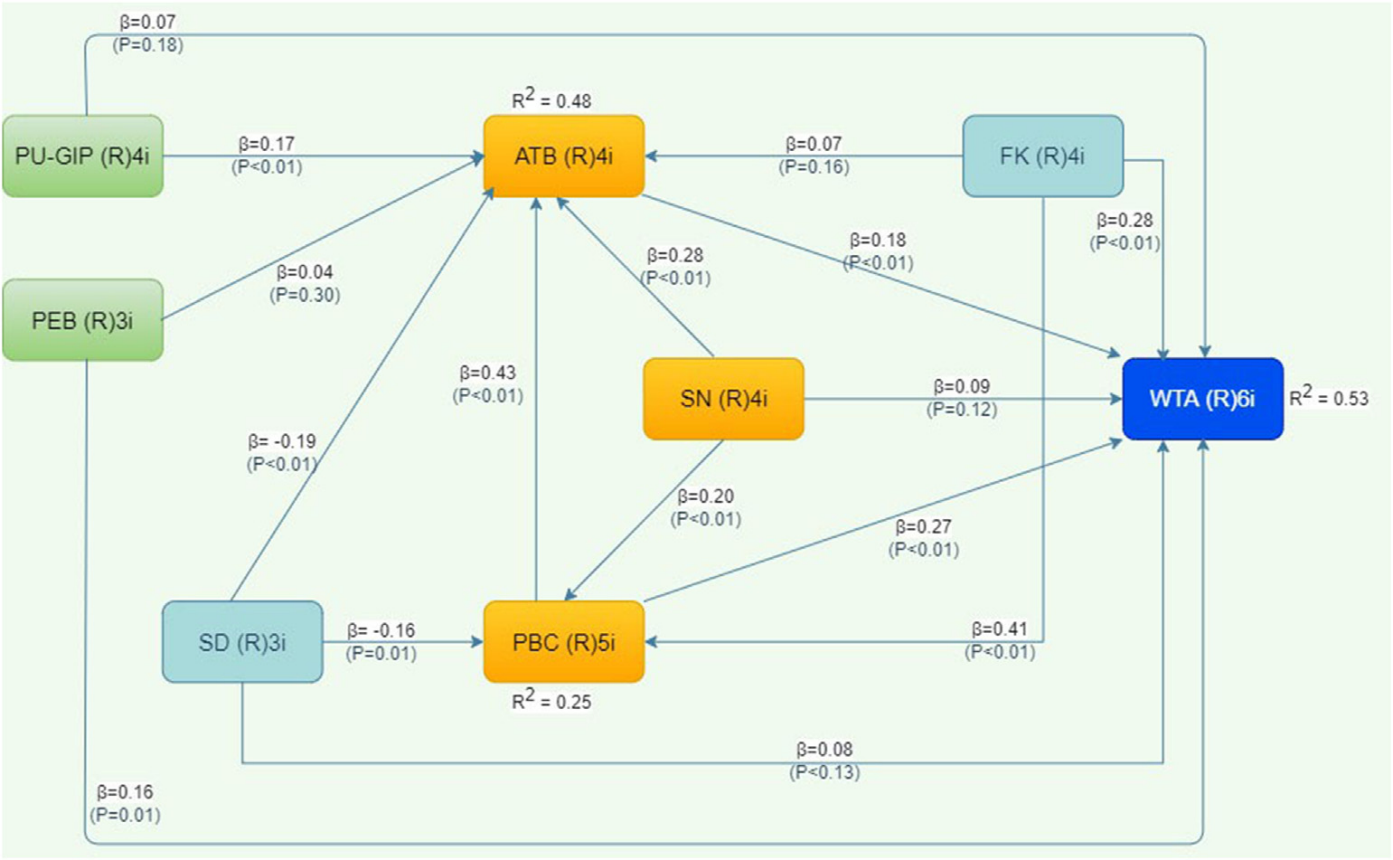
Result of structural model.

The results of hypothesis test is showed in Table 6 given as standardized path coefficients. It implies the significance of the relationships among latent variables. Figure 2 shows the finding of our study. The result shows that attitude toward behavior, perceived behavioral control, perceived economic benefit, farmers’ knowledge and socio demographic characteristics of farmers have a positive effect on the willingness to adopt GIP. The result leads us to accept H1, H3, H8, and H11. Farmers’ knowledge has the greatest direct effect on WTA with the coefficient value 0.281, followed by PBC, ATB and PEB with the coefficient value 0.269, 0.181, and 0.165 respectively. Among the TPB and TAM constructs, PBC has the greatest effect on WTA followed by ATB. However, this study has found that SN is not significantly affecting WTA as the *p*-value is 0.122, then we reject H2.

**Table 6:** The result of structural model and hypothesis test.

Hypothesis	Relationship	Path Coefficient	SE	P-Value	Result
H1	ATB-- > WTA	0.181	0.072	0.006	Accept
H2	SN-- > WTA	0.086	0.074	0.122	Reject
H3	PBC-- > WTA	0.269	0.071	<0.001	Accept
H4	SN-- > ATB	0.280	0.071	<0.001	Accept
H5	SN-- > PBC	0.202	0.072	0.003	Accept
H6	PBC-- > ATB	0.427	0.069	<0.001	Accept
H7	PU-GIP-- > WTA	0.068	0.074	0.178	Reject
H8	PEB-- > WTA	0.165	0.072	0.012	Accept
H9	FK-- > PBC	0.410	0.069	<0.001	Accept
H10	FK-- > ATB	0.073	0.074	0.161	Reject
H11	FK-- > WTA	0.281	0.071	<0.001	Accept
H12	SD-- > PBC	-0.161	0.073	0.014	Accept
H13	SD-- > ATB	-0.188	0.072	0.005	Accept
H14	SD-- > WTA	-0.082	0.074	0.133	Reject
H15	PU-GIP-- > ATB	0.171	0.072	0.009	Accept
H16	PEB-- > ATB	0.039	0.074	0.300	Reject

Notes: ATB = Attitude toward behavior; WTA = Willingness to Adopt; PBC = Perceived behavioral control; SN = Subjective norm; PU-GIP = Perceived usefulness of Geographical Indication practices; PEB = Perceived economic benefit; FK = Famers’ knowledge; SD = Socio demographic.

The inter-relationships between TPB constructs have been shown by the relationship between SN, ATB and PBC. The result of analysis shows that SN has positive and significant effect on ATB and PBC with coefficient values 0.280 and 0.202 respectively. PBC also shows a significant and positive influence on ATB (0.470). Thus, based on the result, the H4, H5, H7 is considered to be accepted.

In regarding the use of the construct of TAM, we divided the construct of perceived usefulness into two variables which is perceived usefulness of geographical indication practices and perceived economic benefit. These two constructs are dimensions and also construct the attitude toward behavior (Pappa et al., 2018). The result shows that only perceived usefulness of geographical indication practices positively and significantly affects ATB (0.171).

Furthermore, regarding the latent variable of farmers’ knowledge and socio demographic characteristics that is added to this model, it has been revealed that farmers’ knowledge demonstrates a significant influence on two TPB constructs which is PBC (H9 is accepted) and WTA. Finally, socio demographic characteristics – consisting of the indicator of age, experience and farm size – demonstrate a negative and significant influence on PBC and ATB. The remaining hypotheses (H2, H7, H10, H14, H16) are unacceptable due to the insignificance issue.

### 5.4 Discussion and implication

This study examines several different factors influencing farmers' intention to accept geographical indications standards using psychological indicators. Referring to several prior studies, which added knowledge and socio-demographic variables, this study seeks to provide a better illustration to explain factors that influence farmers' decisions in adopting geographic indication schemes in Indonesia. The use of the TPB and TAM to determine factors influencing farmers' willingness to adopt GIP as far as we know has never been studied in Indonesia. Thus, the findings of this research will provide new information that supports the use of TPB and TAM constructs as alternative approaches that are suitable for explaining the behavior of coffee farmers in adopting GIP.

The path analysis has shown that among the constructs of TPB and TAM, PBC is the most significant factor influencing farmers’ willingness to adopt GIP followed by attitude toward behavior and perceived economic benefit. This implies that farmers' perception of the ease regarding the GIP is important in affecting farmer decisions to accept GI practices. This result is in line with the theory of TPB (Icek Ajzen, 1991) which revealed the constructs of PBC and ATB is the dominant factor that determines intention. According to Madden et al. (1992), when engaging in a difficult challenge, PBC usually can play in determining behavioral intention. However, in terms of the probability of acceptance, it will increase the probability of accepting new technology if perceived ease to use a new technology by farmers is non-difficult (Saengavut and Jirasatthumb, 2021). Several prior research have similarly found that the most significant role has been played by PBC (TPB construct) or PEOU (TAM construct) in influencing farmers' decisions to embrace a new technology (Bagheri et al., 2019; Borges et al., 2014; Daxini et al., 2019; Pappa et al., 2018; Saengavut and Jirasatthumb, 2021). Whereas, in other studies, PBC did not appear as the dominant effect on farmers’ willingness to adopt such kind of behavior related to technology and system in agricultural research (Govindharaj et al., 2021; Rezaei et al., 2019; Savari and Gharechaee, 2020; Senger et al., 2017). Other TPB and TAM constructs that show a significant relative intention to adopt geographical indication practices are attitude toward behavior and perceived economic benefit with path coefficients 0.181 and 0.165 respectively.

Based on the composite effect of PU and PEB on attitude toward behavior, our empirical study demonstrates that perceived economic benefit influences farmers’ behavior and willingness to adopt geographical indication practices, while in contrast, perceived usefulness of implementing geographical indication code of practices was not. In terms of the relationship between attitude and willingness to adopt GI practices, this study reveals that perceived economic benefit – defined as the economic usefulness perceived by farmers, and or farmers’ perception of economic benefit regarding the GI practices – did not significantly affect attitude toward behavior but shows a significant direct influence on WTA. Different from perceived economic benefit, perceived usefulness of GI practices – as the same as perceived economic benefit, both are an antecedent to attitude toward behavior – had no discernible impact on willingness to adopt geographical indication practices., nevertheless proved a positive relationship on attitude toward behavior. It seems that farmers appear to be most concerned with the financial aspect while considering whether or not to adopt geographical indication practices. Adopting GIP entails higher expenses for the producers, both in terms of fulfilment of requirements and the preparation (Medeiros and Passador, 2015), which are supposed to be compensated by the benefits received. Moreover, there is no guarantee from the certification itself, to gain such a benefit (Medeiros et al., 2016) by implementing geographical indication specification. Therefore, we argue that what is perceived by farmers in terms of the economic benefit – related to the geographical indication specification – influences farmers’ decision to adopt GIP.

Furthermore, we discovered that subjective norms has no significant influence on intention to adopt geographical indicator practices. Subjective norms are related to the influence of social pressure felt by individuals on a certain behavioral intention (Icek Ajzen, 1991). The impact of external pressures like extension workers, farmer groups, and MPIG was not significant in this study. Some of the reasons for this include the farmer's decision to adopt the practice of geographical indications, which is to be impacted strongly by economic issues, as the adoption of GI standards results in additional costs that must be borne by farmers, particularly for GI-compliant post-harvest handling. Several prior studies have shown that the effect of subjective norm on behavioral intention has shown varied results, varying from no significant impact to a significant effect (Venkatesh and Davis, 2000). It is consistent with previous studies in the field of agricultural research, which have produced conflicting results on the effect of external pressure, with individuals not perceiving all significant referents influencing their actions (Borges et al., 2014). The similar results revealed by Rezaei et al. (2019), Verma and Sinha (2018), and Jiang et al. (2018), show a significant influence of SN on ATB and PBC, but did not significantly affect intention. Instead of showing significant direct effect on willingness to adopt, our proposed model demonstrated a significant indirect impact of SN on WTA. The finding implies that ATB and PBC can reflect the effect of subjective norm on willingness to adopt GIP. Furthermore, the findings also show that PBC and ATB were positively influenced by SN. This finding was validated in prior studies (Daxini et al., 2019; Quintal et al., 2010; Verma and Sinha, 2018) and this is consistent with the broader idea that social pressure can influence people to conduct a particular behavior (Bamberg and Möser, 2007). Subjective norms in this study related to the role of MPIG management, government extension officer, and famers’ group who take part in disseminating information related to geographical indication. According to Neilson et al. (2018), proper organizational capacity within MPIG, as well as support in the form of community acknowledgment and acceptance of GIs, have an impact on institutionalizing GIs in the producer community. Therefore, due to a negative effect of subjective norm has been shown in this study, we suggest that MPIG, government extension officers and farmers groups can optimize their role in order to motivate and promote geographical indication to the farmers.

Furthermore, regarding the variable of farmers’ knowledge proposed in this research, the finding shows that knowledge has given a greatest influence on farmers’ willingness to adopt geographical indication based on the standardized path coefficient (Table 6) and standardized total effect (Table 7). The effect of farmers’ knowledge toward the willingness to adopt geographical indication is significant and positive. It plays an important role as the most influential variable affecting willingness to adopt GI. This finding clearly shows that having a high degree of GI knowledge has a greater impact on willingness to adopt GI standards. The similar findings showed that the level of knowledge demonstrated as the most significant variable that gives the total effect towards behavioral intention (Bagheri et al., 2019; Tama et al., 2021). Embedding an understanding about GI code of practice within producers is important in implementing a quality control system (Neilson et al., 2018). Therefore, this study considers farmers’ knowledge and other TPB and TAM constructs to give a better understanding of the farmers’ intention to adopt GIP. Moreover, farmers’ knowledge affects significantly and positively towards PBC that implies the higher the level of farmers’ knowledge about GI concept, the greater the effect on perceived behavior control. This result regarding the importance of knowledge in affecting behavioral intention also highlighted in the prior studies (Armitage and Conner, 2001; Bagheri et al., 2019; Bamberg and Möser, 2007; Tama et al., 2021; Yazdanpanah et al., 2015).

**Table 7:** Standardized total effect.

	PU-GIP	PEB	ATB	PBC	FK	SD	SN
ATB	0.171∗∗	0.039		0.427∗∗∗	0.249∗∗∗	-0.257∗∗∗	0.366∗∗∗
PBC					0.410∗∗∗	-0.161∗	0.202∗∗
WTA	0.099	0.172∗∗	0.181∗∗	0.346∗∗∗	0.436∗∗∗	-0.172∗∗	0.207∗∗

Notes: ∗∗∗ significant at *p*-value < 0.001; ∗∗ significant at *p*-value < 0.01; 1 significant at *p*-value < 0.05.

Finally, this study reveals that the effect of farmers’ socio demographic characteristics on willingness to adopt GIP was insignificant and shows a negative effect, nonetheless it significantly affects farmers’ willingness to adopt GI practices based on the total effects in this proposed model (Table 7). Furthermore, the variable socio demographic negatively and significantly influences other constructs of TPB which are ATB and PBC. This result indicates that the socio-demographic characteristics variable which is formed by the indicator of age, experience and farm size will negatively and significantly influence those two constructs of TPB. A negative effect of socio demographic characteristics on TPB construct can be explained that the lower result on socio demographic will lead the higher intention of farmers to adopt GI practices, the better attitude toward GI practices and the better farmers’ perception on GIP. The finding of the study is supported by several previous studies. In terms of the effect of farm size and perception, Zwitek and SawiDska (2017) proved that farm size has a negative effect on the adoption and perception of greening practice in Poland. Moreover, a study on German farmers’ acceptance behavior toward alley cropping system revealed that farm size negatively affected perceived usefulness although did not show a significant result (Otter and Beer, 2021). According to these prior studies and our finding, it seems that the larger the farm managed by farmer for coffee cultivation will increase the difficulties in adopting GI practices and will cause a negative perception toward GI practices. We argue this situation is related to the availability of capital, labor costs and production cost. The larger farm managed by farmers for cultivation, consequently will cause the higher cost for production.

Another prior study also revealed that demographic variables included age, gender, period of work, and origin district significantly influenced farmers’ satisfaction with a negative relationship (Fernandes et al., 2021), where the indicator of age and period of work showed as a negative predictor. The same result was revealed by Dlamini et al. (2021) and Castillo et al. (2021) where farmers age has a negative influence on perception and behavioral intention to adopt pressurized irrigation technologies. Peng et al. (2020) suggested that personal characteristic factors (age and education) were not significant in influencing farmers’ intention to cultivate wheat in China, most likely because they were getting older. Generally speaking, it can be argued that as farmers get older, their willingness to adopt GI practices decreases. In terms of farmers’ experience, this study indicates that the more experienced the farmers, the more difficult for them to adopt new practices regarding geographical indication. More effort may be needed to target older and more experienced farmers due to they could have a hard time adjusting their habits (Eweoya et al., 2021).

Based on our research findings, we recommend that approaches to promote the practice of geographic indications are carried out by taking into account the socio-demographic factors of farmers. The approach to young farmers is highly necessary because there is a tendency that they have a higher desire to adopt GI practices. In addition, those who involved in promoting GI is also necessary to persuade smallholding farmers to join the GI scheme. More importantly, MPIG organizers, agricultural extension workers, and farmer groups can optimize their role in promoting the GI scheme to the farmers by providing knowledge, information, understanding and training for their better understanding of the GI. The findings have several important policy implications. First, farmers’ knowledge (FK) is critical since it was discovered to have the most direct impact on farmers' willingness to adopt GI schemes of all constructs. Farmers' awareness of GI should be improved through appropriate government policies and programs. GI socialization and awareness building programs could be a government intervention in this case. We also suggest extension agents and MPIG should focus on disseminating information and knowledge related to IG schemes to the farmers. Second, among the TPB and TAM constructs, PBC was observed to have the greatest direct impact on farmers’ willingness to adopt GI schemes. The result shows that farmers’ confidence and ability to comply with GIs’ requirements affect their intention to adopt GI schemes. Therefore, the government and MPIG should provide adequate technical assistance, training, and other support to help farmers increase their ability to produce coffee under GIs’ scheme.

## 6 Conclusion

Based on a combined TPB and TAM model, this study contributes to the enrichment of the previous literatures with regards to how sociodemographic and psychological aspects affecting farmers’ willingness to adopt geographical indication practices (GIP), particularly in developing countries such as Indonesia. The findings of this study indicate that the combined TPB and TAM construct can explain farmers’ behavioral intention to adopt GIP. Applying additional two variables to the suggested framework (farmers’ knowledge and sociodemographic characteristics) can improve the theory's prediction power and accuracy. Moreover, farmers’ knowledge contributes the greatest influence on the willingness to adopt GIP followed by perceived behavioral control, attitude toward behavior and perceived economic benefit. External pressure represented by a subjective norm in this model, has no influence on farmers’ willingness to adopt GIP. Embedding understanding and knowledge about geographical indication (GI) code of practice towards producers is important in implementing GI schemes. This suggests that proactive efforts are needed to promote GI schemes to the farmers by concerned stakeholders such as GI association (MPIG), agricultural extension officers, and farmers’ groups. The strategy to promote the GI scheme can take into account the results of this study, where the sociodemographic factors (i.e., age, farm size and experience) showed a negative influence on the TPB construct. We recognize the study’s limitations, realizing the difficulty of using representative random sampling due to the lack of a complete list of the total population to be examined under Covid-19 pandemic condition. Therefore, generalization in this study could be another issue to be considered for the next study.

## Declarations

### Author contribution statement

Pandu Laksono: Conceptualization and designing the experiments; data curation; investigation; analysis tools and data; analyzing and interpreting the data; writing the original draft.

Irham: Conceptualization and designing the experiments; analyzing and interpreting the data; writing – review and editing.

Jangkung Handoyo Mulyo: Conceptualization and designing the experiments; analyzing and interpreting the data; writing – review and editing.

Any Suryantini: Conceptualization and designing the experiments; analyzing and interpreting the data; writing – review and editing.

### Funding statement

This research did not receive any specific grant from funding agencies in the public, commercial, or not – for – profit sectors.

### Data availability statement

Data will be made available on request.

### Declaration of interest statement

The author declares no conflict of interest.

### Additional information

No additional information is available for this paper
